# A Partially Randomized Patient Preference Trial to Assess the Quality of Life and Patency Rate After Minimally Invasive Cardiac Surgery-Coronary Artery Bypass Grafting: Design and Rationale of the MICS-CABG PRPP Trial

**DOI:** 10.3389/fcvm.2022.804217

**Published:** 2022-04-25

**Authors:** Yichen Gong, Xiaoxiao Wang, Nan Li, Yuanhao Fu, Hui Zheng, Ye Zheng, Siyan Zhan, Yunpeng Ling

**Affiliations:** ^1^Department of Cardiac Surgery, Peking University Third Hospital, Beijing, China; ^2^Research Center of Clinical Epidemiology, Peking University Third Hospital, Beijing, China; ^3^Department of Epidemiology and Biostatistics, School of Public Health, Peking University, Beijing, China

**Keywords:** MICS-CABG, patients' preference, quality of life, patency rate, trial, minimally invasive, surgical procedures, SF-36 score

## Abstract

**Background:**

Minimally invasive cardiac surgery-coronary artery bypass grafting (MICS-CABG) has emerged as a safe alternative to standard cardiac surgery. However, treatment preferences can decrease the generalizability of RCT results to the clinical population (i.e., reduce external validity) and influence adherence to the treatment protocol and study outcomes (i.e., reduce internal validity). However, this has not yet been properly investigated in randomized trials with consideration of treatment preferences.

**Study Design:**

In this study, patients with a preference will be allocated to treatment strategies accordingly, whereas only those patients without a distinct preference will be randomized. The randomized trial is a 248-patient controlled, randomized, investigator-blinded trial. It is designed to compare whether treatment with MICS-CABG is beneficial in comparison to CABG. This study is aimed to establish the superiority hypothesis for the physical component summary (PCS) accompanied by the non-inferiority hypothesis for overall graft patency. Patients with no treatment preference will be randomized in a 1:1 fashion to one of the two treatment arms. The primary efficacy endpoints are the PCS score at 30 days after surgery and the overall patency rate of the grafts within 14 days after surgery. Secondary outcome measures include the PCS score and patency rate at different time points. Safety endpoints include major adverse cardiac and cerebrovascular events, complications, bleeding, wound infection, death, etc.

**Conclusions:**

This trial will address essential questions of the efficacy and safety of MICS-CABG. The study will also address the impact of patients' preferences on external validity and internal validity.

## Introduction

Coronary artery bypass grafting (CABG) has always been the gold standard for surgical treatment of coronary artery disease (CAD), especially for patients with high SYNTAX scores, left main (LM) coronary disease, and diabetes mellitus (1–3). In clinical practice, traditional sternotomy CABG results in trauma and slow post-operative recovery, especially for patients with obesity, women, patients with diabetes and patients with bilateral internal mammary artery (BIMA) involvement, who may face the risk of deep sternal wound infection (DSWI) (4–6). In addition, because of the fear of thoracotomy surgery, some patients delay treatment. Thoracotomy also brings psychological trauma to patients after surgery, which will also increase their mortality (7). Minimally invasive cardiac surgery-CABG (MICS-CABG) is a new surgical strategy that performs CABG through a left anterolateral thoracic incision. This procedure can effectively reduce bleeding and avoid the risk of sternal instability and DSWI (8). Theoretically, MICS-CABG can result in faster rehabilitation and better quality of life of patients (9) and is expected to become a surgical selection for CAD.

Although some cohort studies have reported good perioperative safety and the short-term follow-up results and graft patency are similar to those of traditional CABG (10), the worldwide percentage of MICS-CABG procedures is relatively low. By avoiding sternotomy, patients recover significantly faster in the early post-operative period. However, patients' perceptions of wellness after minimally invasive approach lack higher-level clinical evidence. A prospective randomized controlled trials (RCT) is needed to compare the quality of life between MICS-CABG and sternotomy-CABG patients. In addition, the quality of anastomosis and graft patency are crucial factors that determine the long-term prognosis of patients. MICS-CABG performs vascular anastomosis through a limited narrow space, and whether this procedure can achieve a satisfactory graft patency rate is unclear. Therefore, the efficacy of MICS-CABG would be insufficient as a single primary endpoint (quality of life) and should be accompanied by an additional co-primary endpoint (overall patency rate).

It is worth pointing out that some patients often show a greater preference for minimally invasive procedures after being informed that CABG can be performed with a small incision. Therefore, patients may refuse to participate in RCTs to avoid sternotomy surgery, which results in limiting extrapolation of the study results (i.e., reduced external validity). In addition, the existence of patients' preferences may lead to reduced compliance, resulting in increased loss of follow-up, thus affecting the internal validity of the study results. Furthermore, patients' preferences may have an impact on the assessment of quality of life, which is the primary outcome of the study. To effectively solve the above problems, the present study adopted a partially randomized patient preference (PRPP) trial. This randomized patient preference trial is an RCT and preference cohort combined. Patients with a preference will be allocated to treatment strategies accordingly, whereas only those patients without a distinct preference will be randomized. In addition, they will be followed up using the same method. And systematic reviews (11) addressing influence of PRPP design on validity concluded that PRPP design could increase external validity without compromising the internal validity compared with RCTs. The study is designed to compare whether treatment with MICS-CABG is beneficial in comparison to CABG and is aimed to establish the superiority hypothesis for quality of life accompanied by the non-inferiority hypothesis for overall patency rate.

## Materials and Methods

**Figure 1:**
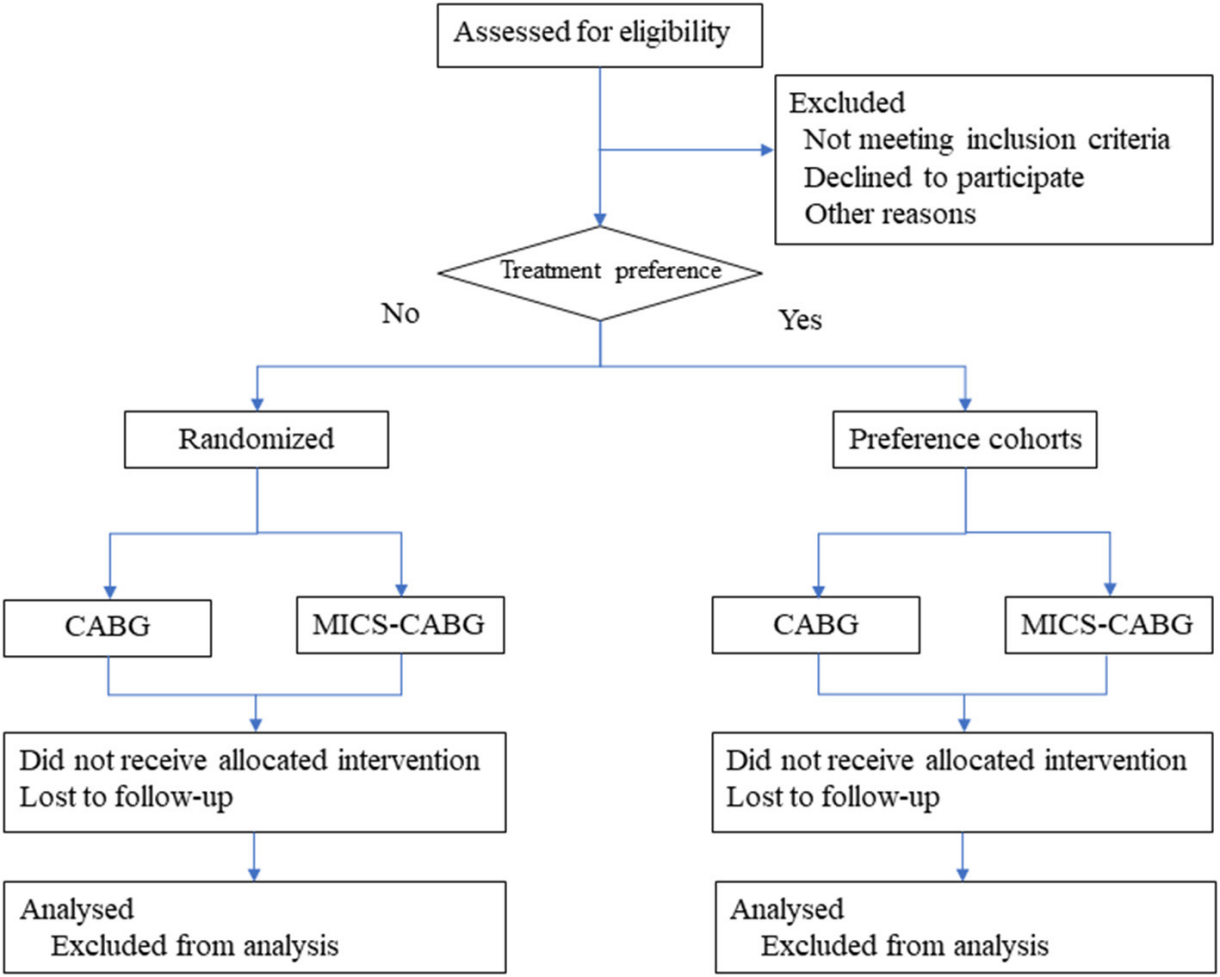
Patient flow diagram.

This randomized patient preference trial had been registered at ClinicalTrials.gov, identifier: NCT04795193. The trial has been reviewed and approved by Peking University Third Hospital Medical Science Research Ethics Committee. Any modifications to the protocol will be agreed upon by the ethics committee. The researcher will obtain informed consent from eligible participants. The study center, the Department of Cardiac Surgery at Peking University Third Hospital, conducts an estimated 500 coronary artery bypass surgeries annually (MICS-CABG accounts for 60%). Inpatients from October 2021 to December 2023 will be selected for the eligibility evaluation process. The Patient flow diagram is displayed in Figure 1.

### Study Population

#### Inclusion Criteria

① Patients aged 25–85 years who were scheduled for cardiac surgery; ② Symptomatic significant multivessel coronary artery disease including left main stem stenosis.

#### Exclusion Criteria

① Unstable preoperative hemodynamic status (vasoactive drugs such as dopamine, epinephrine or norepinephrine to maintain blood pressure, or an intra-aortic balloon pump [IABP] is implanted preoperatively) or requiring emergency surgery. ② Severe emphysema, hypoxemia [postbronchodilator forced expiratory volume in 1 s (FEV1)/forced vital capacity (FVC) < 70% and FEV1% predicted < 50% or partial pressure of oxygen (pO_2_) < 60 mmHg or partial pressure of carbon dioxide (pCO_2_) > 40 mmHg without oxygen therapy]. ③ Old extensive myocardial infarction without a viable myocardium based on isotope and echocardiography examination, significant cardiac enlargement [cardiothoracic ratio > 0.75, EF < 30%, left ventricular diastolic diameter (LVDd) > 60 mm, left ventricular aneurysm or severe arrhythmia]. ④ Severe pleural adhesion, chest deformity, or previous thoracic radiotherapy. ⑤ Previous thoracotomy surgery. ⑥ Simultaneous valve or other cardiac surgery. ⑦ Planned cardiopulmonary bypass surgery. ⑧ Poor condition of the distal coronary artery [diffuse stenosis, chronic total obstructive lesion with severe calcification or inability to match the graft due to a small diameter (<1.0 mm)]. ⑨ Intolerance to surgery in combination with the following complications: Terminal cancer, uncontrolled infection, bleeding, severe brain injury, infarction or bleeding, multiple organ failure and other major organ dysfunction such as severe liver dysfunction or severe congestive heart failure.

#### Baseline

Baseline indicators include age, sex, height, weight, BMI, previous disease history (diabetes mellitus, hyperlipidemia, hypertension, smoking, renal insufficiency, myocardial infarction and previous history of invasive therapy, such as CABG, PCI and other cardiac surgery), angina classification (Canadian Cardiovascular Society classification, CCS), cardiac function classification (New York Heart Association, NYHA classification), laboratory testing (serum level of creatinine, cholesterol, LDL-C, BNP, CK-MB, troponin, etc.), preoperative examination (cardiac ultrasound, pulmonary function, blood gas analysis, electrocardiogram, peripheral artery ultrasound, chest CT, etc.), mortality calculation by EuroScore II scale, SF-36 and Seattle Angina Questionnaire (SAQ) scores, the SYNTAX score and the anticipated bypass strategy.

### Intervention and Surgical Procedure

The surgeons performing the procedure, who have received formal training, will be responsible for obtaining informed consent. They are required to fully inform their patients about the benefits and harms of MICS-CABG and sternotomy CABG. Patients will decide whether to participate in the trial, and they can freely withdraw (discontinue participation) at any time during the trial. Their refusal and withdrawal will not negatively impact the therapeutic relationship.

Off-pump CABG were the preferred surgical strategy for both groups. All surgeons are qualified surgeons with rich experience in cardiac surgery and have accumulated more than 500 cases of CABG and more than 60 annual cases of CABG. The surgeons who will perform the MICS-CABG procedure should have performed ≥100 multivessel MICS-CABG cases and have >3 years of experience with MICS CABG.

The angiogram will be assessed by at least two surgeons, and the surgical plan (the number, material and configuration of the graft) will be determined and recorded before the operation. All coronary lesions with distal vessel diameters >1.5 mm will be treated with grafts. The actual bypass procedure will be recorded and compared with the preoperative plan.

Left internal mammary artery (LIMA)-left anterior descending branch (LAD) bypass is the first choice for all patients. Off-pump CABG is performed routinely in both sternotomy and MICS-CABG surgery. Cardiopulmonary bypass will be prepared for all patients, and its use will be determined according to the intraoperative conditions. The exposure of target coronary artery territories is realized by the stabilizer of HT-KD or Medtronic. Surgeons will choose the bypass strategy according to their experience, including whether to use the bilateral internal mammary artery and radial artery for complete arterial bypass, whether to perform ascending aortic anastomosis, and the sequential graft strategy.

Off-pump CABG (OPCABG) will be performed in a traditional surgical method. Gauze will be used to protect the sternum when the retractor is used during the sternotomy surgery. A skeletal strategy will be used to prevent DSWI when obtaining the bilateral internal mammary arteries.

MICS-CABG will be performed through a left anterolateral incision. A bilateral femoral arteriovenous assessment will be conducted preoperatively, and femoral arteriovenous bypass could be used to establish cardiopulmonary bypass in cases of circulatory instability or other emergency conditions during the operation. Double lumen endotracheal intubation will be used for anesthesia, and a single-lung ventilation strategy will be employed according to the surgeons' decision. The internal mammary artery, coronary artery and ascending aorta will be exposed by a special retractor. The epicardial and apical stabilizer will be used to expose the target coronary vessel and accomplish the anastomosis. If circulatory instability or severe bleeding occurs during the operation, the surgeon may choose to perform extracorporeal circulation *via* femoral arteriovenous bypass or to switch to thoracotomy, depending on his or her experience.

### Randomization

Participants who have no treatment preference will be randomized in the usual way. Randomization will be performed by a computer-generated random number list prepared by independent statisticians with no clinical involvement in the trial. The block randomization sequence is created using SAS 9.4 software (PROC PLAN) and a 1:1 allocation. The block sizes will not be disclosed to ensure concealment. After the physicians responsible for recruitment obtain eligible patient consent, the research nurse will contact an online, central randomization service by a secure computer for allocation consignment. The research nurse will then provide information about the treatment allocation to the intervention physicians.

### Blinding

An assessor blinded to the allocation will conduct telephone interviews to assess the quality of life of the patients. The patency rate of the grafts will be assessed with coronary angiography by two independent specialist physicians blinded to the allocated arm. In addition, data analysts outside the research team will be kept blind to the allocation. Because patients and physicians allocated to the intervention group will be aware of the treatment allocation, they will be asked not to disclose the allocation status.

### Outcomes

#### Primary Outcome and Definitions

SF-36 PCS (physical component summary, PCS) score at 30 days after surgery: The SF-36 is a concise health questionnaire developed by the Boston Health Institute in the United States that has been widely used in the measurement of quality of life in the general population, the evaluation of clinical trial effects and the evaluation of health policy. The SF-36 is a concise health test that features Physical Functioning (PF), Roe-Physical (RP), Bodily Pain, General Health (GH), Vitality (VT), Social Functioning (SF), Roe-Emotional (Emotional), and Mental Health (MH) functioning and comprehensively summarizes the quality of life of the respondents across 8 aspects. The PCS and mental component summary (MCS) can be calculated from the eight abovementioned indicators with different weights. The PCS score is selected as the primary outcome. The surgical methods of the patients will be hidden with the follow-up researchers when they conduct telephone or online follow-up according to the questionnaire. The follow-up results will be entered into the database by another researcher.

Patency rate of total grafts within 14 days after surgery: The patency rate of the grafts will be assessed *via* reexamination by coronary angiography or coronary CTA within 14 days after surgery. Coronary angiography is preferred for all subjects, and coronary CTA is used for patients with renal insufficiency, intolerance to contrast agent, and other conditions that doctors consider unsuitable for angiography. Fitzgibbon grading criteria are used to evaluate patency, as follows: grade A: bridge vessels are patent without stenosis or stenosis diameter <50%; grade B: bridge vessels have a stenosis diameter of 50–99%; grade O: bridge vessels exhibit complete occlusion. Fitzgibbon grade A indicates patency. The evaluators will be blinded, the surgical method and name of the patients will be hidden, and the patency of the bridging vessels will be assessed by angiography or CT.

#### Secondary Outcomes and Definition

SF-36 PCS and MCS scores: The PCS and MCS scores will be evaluated by telephone or online at 7 days, 3 months, 6 months, and 1 year after surgery to measure the physiological and psychological status of patients.

Patency rate of different target vessels within 14 days after surgery: patency rate of LAD, of right coronary artery (RCA), of circumflex branch (LCX) and of diagonal branch (D).

Patency rate of different types of grafts within 14 days after surgery: patency rate of LIMA, of RIMA, of saphenous vein graft (SVG) and of radial artery (RA).

Patency rate of the grafts at 1 year after surgery: The patency rate of the grafts will be assessed by coronary CTA at 1 year after surgery.

Length of incubation: defined as the time of assisted respiratory ventilation after surgery.

Hospitalization cost: total hospitalization cost of each patient.

Length of post-operative hospitalization time: total post-operative hospitalization time of the patients (unit: days).

Completeness of revascularization (CR) index: the number of grafts performed divided by the number of grafts needed (number of graftable vessels with angiographically significant stenoses).

#### Safety Endpoints

All safety aspects will be monitored by a Data Safety Monitoring Board (DSMB) consisting of 2 cardiologists and 1 statistician. Safety will be assessed in terms of bleeding, infection, secondary surgery, ischemic vascular complications, and deaths.

Intrahospital RBC transfusion volume: the amount of red blood cells (U) to be transfused during hospitalization. Indications for blood transfusion include hemoglobin <90 g/L, severe intraoperative or post-operative active bleeding, or other blood transfusion considered necessary by the surgeons.

Wound infection rate: defined as wound dehiscence, effusion and secondary debridement and suture within 3 months after surgery.

Re-exploration for bleeding or other causes (not including wound-related causes): defined as the requirement to return to the operating room for reopening of sternotomy or MICS CABG incision for any reason, such as bleeding, post-operative acute myocardial ischemia and unexplained circulatory instability. Debridement for DSWI or infection of anterior-lateral wounds will be excluded.

Major adverse cardiac and cerebrovascular events (MACCEs): Composite endpoint of all-cause death, non-fatal myocardial infarction, stroke, and target vessel ischemia-driven repeated revascularization (TVR). The patients will be followed-up at 1, 6, 12, 24, 36, and 60 months after surgery, and the first occurrence of MACCE will be recorded.

### Follow-Up Procedure

We will maintain interest in the study through materials and mailings. The researchers will conduct follow-up by telephone, online websites or mobile apps, and the patients' surgical group information will be concealed from them; instead, only the list of patients' names will be supplied. Follow-up procedure is displayed in Table 1.

**Table 1:** Follow-up plan.

	**Period**													
	**Enrollment**	**Allocation**	**Surgery**	**Post-surgery**										
	**-16 M**	**0**		**Hospital discharge**	**7 D**	**14 D**	**1 M**	**3 M**	**6 M**	**12 M**	**24 M**	**36 M**	**48 M**	**60 M**
Timepoint*														
**Enrollment:**														
Eligibility screen	√													
Informed consent	√													
Allocation		√												
**Interventions:**														
MICS-CABG			√											
Sternotomy CABG			√											
**Assessments:**														
Demographic baseline	√													
Clinical baseline	√													
Preoperative examination	√													
Preoperative coronary angiography information	√													
Prediction of the surgical strategy		√												
Surgical information			√											
Post-operative examination				√										
Blood transfusion				√										
Post-operative complication				√										
SF-36 scale	√				√		√	√	√	√				
SAQ scale	√													
Patency of grafts (by CT or angiography)						√				√				
Length of incubation				√	√									
Hospitalization time				√	√									
Wound infection				√	√		√	√						
MACCE				√	√		√	√	√	√	√	√	√	√
Death				√	√		√	√	√	√	√	√	√	√
Smoke	√						√	√	√	√	√	√	√	√
Drug therapy	√						√	√	√	√	√	√	√	√
Other related primary disease (diabetes, hypertension, perivascular disease, etc.)	√						√	√	√	√	√	√	√	√
	√						√	√	√	√	√	√	√	√

### Sample Size Calculation

The sample size was calculated on the basis of the superiority hypothesis for PCS accompanied by the non-inferiority hypothesis for the overall patency rate of the grafts. The margin of superiority was set at 2. The margin was based on clinically and statistically important differences as well as ethical criteria, cost, and feasibility. The sample size of 172 patients (86 in each group) was calculated to be sufficient (with a two-sided 95% CI and 90% power) to establish the superiority hypothesis, when we assume there is a difference of 7 between groups with an estimated standard deviation of 10. Given an anticipated dropout rate of 10%, the total sample size required is 190 (95 in each group).

The margin of non-inferiority was 6%. Based on an expected overall patency rate of 96% and 96% in MICS-CABG and sternotomy CABG, we calculated that a sample of 496 grafts (248 in each group) is necessary to give 90% power to establish the non-inferiority hypothesis (a one-sided type 1 error of 2.5%), assuming a 0% reduction in MICS-CABG. The sample size calculation allows for 10% loss to follow-up. Based on the fact that an average of 2–3 grafts are needed for each patient, a sample size of 248 patients (124 in each group) is needed. Therefore, this study would require 248 patients in total with equal allocation to two arms.

### Data Collection and Management

The researchers will be centrally trained in the study requirements to promote data quality. A Data Monitoring Committee (DMC) independent of the study organizer has been established to periodically review the accumulating data. All study-related information will be stored securely at the study site. All participant information will be stored in article questionnaires and electronic forms with limited access.

All clinical indicators (laboratory tests, imaging data, and questionnaires, etc.) will be stored in two ways: the EDC system and article document collection. Case data will be kept at least 5 years after the end of the study to ensure the traceability of all data. Data collection and outcome judgments will be conducted by professional doctors with more than 5 years of clinical experience. In accordance with the experimental manual, all the researchers who collected data and surveyed the questionnaire were trained. In this study, the EDC system will be used to provide logical monitoring of key data, set up range values and prompt outliers. The Data Management Committee will check the accuracy of the data every quarter.

### Statistical Analysis

Statistical analyses will be performed using IBM SPSS Statistics for Windows, version 26.0 (IBM Corp., Armonk, N.Y., USA). First, a descriptive analysis will be conducted. Normally distributed continuous variables will be expressed as the mean and standard deviation, and non-normally distributed continuous variables will be expressed as the median and interquartile range (IQR). Categorical variables will be expressed as percentages.

The primary efficacy endpoint will be assessed in the per protocol population and the intention-to-treat (ITT) population. Statistical significance is needed for both primary endpoints. Therefore, no formal adjustment of the significance level of the elementary hypothesis tests is necessary. For the primary efficacy endpoint PCS at 1 month after surgery, the superiority of MICS-CABG to OPCABG could be claimed if the lower limit of the 95% CI (for the difference in PCS scores between groups) is >2. For the other coprimary efficacy endpoint, namely, the overall patency rate at 2 weeks after surgery, non-inferiority of MICS-CABG to OPCABG could be claimed if the lower limit of the 95% CI (for the difference in patency rate between groups) is >-6%. Subgroup analysis and covariate-adjusted effect size were used to explore the effect of “use of BIMA, sequential vs. single anastomoses” on results, if applicable.

To explore the impact of patients' preference on external validity, the participation rate and the randomization refusal rate will be analyzed. The differences in baseline characteristics between the random cohort and the preference cohort will be compared to assess if a specific patient group has accepted randomization. To explore the impact of patients' preference on internal validity, the proportion of patients lost to follow-up will be analyzed. To explore the impact of patients' preference on primary outcomes, we will examine treatment-specific differences between the preference and randomization groups. In addition, we will make comparisons (1) between randomized MICS-CABG and preference MICS-CABG and (2) between randomized sternotomy CABG and preference sternotomy CABG to explore the impact of preference on outcome assessment.

The chi-squared test will be used to examine the differences in binary secondary outcomes (e.g., graft patency rate at different time points and secondary surgery). Kaplan-Meier survival analysis will be employed for timed endpoints such as MACCE. The *t*-test and Mann–Whitney *U*-test will be used to examine the differences in continuous secondary outcomes (e.g., SF-36 at different time points, mechanical ventilation time, hospitalization costs and post-operative hospital stay). The level of significance is set at α = 0.05.

## Discussion

### Safety and Quality of Life in MICS-CABG

As a result of the development of new retractors and minimally invasive surgical instruments, MICS-CABG technology has developed rapidly in the last decade. Although MICS-CABG can technically achieve complete revascularization and total aerial revascularization (12), if the surgical indications are not properly evaluated, it will increase the difficulty and risk of surgery.

MICS-CABG surgery is performed through a narrow space. If emergency conditions, such as major arterial injury, bleeding or acute myocardial ischemic attacks, such surgery will be very dangerous. Therefore, preparation for transfer to thoracotomy and femoral arteriovenous cardiopulmonary bypass should also be routinely considered. The MICS-CABG procedure should be carefully selected for patients with specific conditions (thoracic deformity, thoracic adhesion, severe calcification of the ascending aorta, severe stenosis of the femoral artery, severe respiratory insufficiency, severe obesity, and severe cardiac insufficiency). The percentage of MICS-CABG procedures worldwide is relatively low, and no published randomized controlled trials have been conducted to evaluate its clinical efficacy and safety.

In this study, the physiological function score in the SF-36 score of patients' qualities of life at 30 days after surgery will be taken as the primary outcome to verify whether MICS-CABG can truly improve the quality of life and recovery speed of patients. MICS-CABG is considered to reduce surgical trauma due to the small incision and the avoidance of sternotomy, and the wound healing time is significantly shorter than that in sternotomy. MICS-CABG seems to increase the rehabilitation speed and improve the quality of life of patients after surgery. However, some MICS-CABG patients feel obvious pain in the early post-operative period and even more severe pain than that after sternotomy surgery, which can result in restrictive respiratory movement, weakness of expectoration and delayed rehabilitation. This outcome may be related to traction of the intercostal nerve that causes intercostal nerve injury; on the other hand, post-operative respiratory insufficiency may occur due to intermittent single-lung ventilation during MICS-CABG surgery. Therefore, through an RCT, this study will investigate the influence of MICS-CABG on early post-operative quality of life.

Another primary outcome is graft patency as assessed by coronary angiography within 14 days after surgery. MICS-CABG procedure employs a single small intercostal incision and this procedure has inherent challenges and difficulties. Graft patency associated with the MICS-CABG procedure is related to the quality of the surgical technique. Graft patency in the early post-operative period is less affected by other confounding factors (such as obesity, complications, LDL-C level, smoking, pharmacotherapeutics, etc.), and is a primary determinant of the quality and efficacy of this approach. Graft patency within 30 days after surgery has been used to examine the quality of this minimally invasive means. According to our analysis, most patients are discharged to home between 5 and 10 days after surgery. Therefore, angiography was used to assess graft patency within 14 days after surgery and defined as a primary outcome.

The SF-36 score at each post-operative time point, which included two aspects of the PCS and MCS, was selected as one of the secondary outcomes to evaluate the rehabilitation process of patients. The incidence of MACCE, the 1-year post-operative graft patency rate and other perioperative indicators were collected to explore whether there were differences between MICS-CABG and CABG in other safety aspects. The advantages and limitations of MICS-CABG will be assessed from two aspects of rehabilitation speed and safety in this study.

### Significance of Patients' Preference and the PRPP Design

RCTs are the gold standard for evaluating the efficacy and safety of treatment. The effect of MICS-CABG on patients' quality of life and graft patency can be assessed by RCTs. However, patient preference exists in the process of revascularization treatment of CAD. For example, patients tend to undergo PCI rather than CABG, although the high risk (12-month post-operative high incidence of the composite endpoint of death, stroke, myocardial infarction and repeat revascularizations) of the PCI strategy for multivessel CAD is clearly quoted to them. Patients are more likely to choose minimally invasive treatment (13, 14). Furthermore, it has been shown that patients' preferences impact the evaluation of some clinical outcomes, such as MACCE, which is often used as an endpoint of cardiovascular clinical trials. Patients' preference choices may also have an impact on their compliance (drug compliance and improvement of living habits) (15), which are closely related to the prognosis of CAD. Respecting the patient's propensity may reduce perioperative anxiety and depression, which can influence the post-operative quality of life in both psychological and physical dimensions (16). Therefore, we can conclude that preference could impact the evaluation of outcomes and that ignoring it may reduce the internal authenticity of the study.

Evidently, it is unpredictable which type of CABG will be performed in RCTs; patients who have obvious preferences might have refused to participate in research or randomization. Patients who reject doctors' suggestions for surgical treatment are not uncommon, and this proportion is as high as 30–50% in CABG surgery (17). Because of the fear of sternotomy surgery, some patients even abandon treatment and decide to be discharged. Exiting or changing the intervention of the study will reduce the external authenticity, which affects the extrapolation of results. Currently, the guideline also recommends that physicians consider patients' preferences and make treatment decisions with them. Therefore, the RCT could be an inappropriate design especially for an unblinded trial in which it can be foreseen that patients' preference will be a prominent factor, for example, in trials comparing treatments of significant different nature. PRPP design has been developed to address such problems. In PRPP design, patients with strong preferences are offered their treatment of choice, while those without strong preferences are randomized in the conventional fashion. Clinical data will be collected for all patients, whether they are randomized or not. And PRPP design has certain advantages. First, patients with a distinct preference will be included in the study, improving external validity; second, as only the remaining indifferent patients will be included in the RCT cohort of a PRPP, this RCT cohort can be considered as the true gold standard for internal validity. In conclusion, PRPPs seem to be a reliable alternative for RCTs, especially in trials comparing treatments of vastly different nature or using patient-centered outcomes. In addition, in the era of patients becoming more active participants in research, the use of PRPPs would increase.

## Data Monitoring Committee

This study will be monitored by the DMC, which is established by the foundation sponsor. The DMC is independent of the research group and consists of 1–2 statistical experts and 1–2 clinical experts. The DMC does not usually have executive power; rather, it communicates the outcome of its deliberations to the sponsor. The DMC is responsible for verifying the protocol modification, project process, serious adverse events (SAEs), informed consent forms, etc., and checking the traceability of all data. The DMC will conduct monitoring 1–2 times during the period of recruitment and provide monitoring reports.

## Interim-Analyses and Termination of the Study

No interim analysis will be conducted in this study, and the occurrence of SAEs (MACCEs and death) will be monitored through the Electronic Data Capture (EDC) database. The study will be discontinued when more than 6 deaths or 15 MACCE cases occur in the MICS-CABG randomized group.

## Ethics Statement

The studies involving human participants were reviewed and approved by Peking University Third Hospital Medical Science Research Ethics Committee. The patients/participants provided their written informed consent to participate in this study. Trained Research Residents will introduce the trial to patients, who will be shown a pamphlet or poster regarding the different surgical procedures of the trial. Patients will also receive information sheets. Research Residents will discuss the trial with the patients and tell them the risks and benefits with informed consent. Patients will then be able to have an informed discussion with the participating consultant. Research Residents will obtain written consent from patients willing to participate in the trial. All information sheets, consent forms, pamphlets and posters will be written in Chinese and translated into English when required.

## Author Contributions

YL conceived of the study. YG and XW initiated the study design, are responsible for the drafting, editing of the article, and its final contents. SZ and NL provided statistical expertise in clinical trial design. YF, HZ, and YZ helped with implementation. All authors contributed to refinement of the study protocol and approved the final manuscript.

## Funding

This study was supported by Capital's Funds for Health Improvement and Research, China (Fund No. CRF 2020-2-4096), the National Natural Science Foundation of China (Project Numbers: 82101264, 81701067), Peking University Medicine Fund of Fostering Young Scholars' Scientific, Technological Innovation (Project Number: BMU2021PYB039), Foundation of Beijing Cardiovascular Disease Prevention & Treatment Association (Project Number: XXGFZ202101) and Clinical Cohort Construction Program of Peking University Third Hospital (Project Number: BYSYDL2019016). The funding sources had no role in the design of this study and will not have any role during its execution, analyses, interpretation of the data, or decision to submit results.

## Conflict of Interest

The authors declare that the research was conducted in the absence of any commercial or financial relationships that could be construed as a potential conflict of interest.

## Publisher's Note

All claims expressed in this article are solely those of the authors and do not necessarily represent those of their affiliated organizations, or those of the publisher, the editors and the reviewers. Any product that may be evaluated in this article, or claim that may be made by its manufacturer, is not guaranteed or endorsed by the publisher.
