# Correction: A partially randomized patient preference trial to assess the quality of life and patency rate after minimally invasive cardiac surgery-coronary artery bypass grafting: design and rationale of the MICS-CABG PRPP trial

**DOI:** 10.3389/fcvm.2025.1713150

**Published:** 2025-10-31

**Authors:** Yichen Gong, Xiaoxiao Wang, Nan Li, Yuanhao Fu, Hui Zheng, Ye Zheng, Siyan Zhan, Yunpeng Ling

**Affiliations:** ^1^ Department of Cardiac Surgery, Peking University Third Hospital, Beijing, China; ^2^ Research Center of Clinical Epidemiology, Peking University Third Hospital, Beijing, China; ^3^ Department of Epidemiology and Biostatistics, School of Public Health, Peking University, Beijing, China

**Keywords:** MICS-CABG, patients' preference, quality of life, patency rate, trial, minimally invasive, surgical procedures, SF-36 score

A Correction on A partially randomized patient preference trial to assess the quality of life and patency rate after minimally invasive cardiac surgery-coronary artery bypass grafting: design and rationale of the MICS-CABG PRPP trial By Gong Y, Wang X, Li N, Fu Y, Zheng H, Zheng Y, Zhan S and Ling Y (2022). Front. Cardiovasc. Med. 9:804217. doi: 10.3389/fcvm.2022.804217

At the inception of this RCT, the study was registered under the number NCT04795193. Due to adjustments in team responsibilities and unfamiliarity with clinical trial registration rules, a revised protocol was mistakenly submitted as a separate registration under NCT05104320. This registration number was then used during the submission of the protocol manuscript. Upon subsequent verification of the RCT, we discovered this inconsistency and intend to consolidate the information from both registration entries into the original registration under NCT04795193, while withdrawing the duplicate registration NCT05104320. The registration number “NCT05104320” in the published article should be replaced with “NCT04795193.”

A correction has been made to the sentence in **section Materials and Methods**, paragraph 1:

“This randomized patient preference trial had been registered at ClinicalTrials.gov, identifier: NCT04795193.”

The original version of this article has been updated.

